# A Cuprous Oxide Thin Film Non-Enzymatic Glucose Sensor Using Differential Pulse Voltammetry and Other Voltammetry Methods and a Comparison to Different Thin Film Electrodes on the Detection of Glucose in an Alkaline Solution

**DOI:** 10.3390/bios8010004

**Published:** 2018-01-06

**Authors:** Yifan Dai, Alireza Molazemhosseini, Kevin Abbasi, Chung Chiun Liu

**Affiliations:** 1 Department of Chemical and Biomolecular Engineering and Electronics Design Center, Case Western Reserve University, 10900 Euclid Ave, Cleveland, OH 44106, USA; yxd176@case.edu; 2 Dipartimento di Chimica Materiali ed Ingegneria Chimica “Giulio Natta”, Politecnico di Milano, Via Mancinelli 7, 20131 Milano, Italy; axm1058@case.edu; 3 Swagelok Center for Surface Analysis of Materials (SCSAM), Case Western Reserve University, 10900 Euclid Ave, Cleveland, OH 44106, USA; kxa244@case.edu

**Keywords:** cuprous oxide, non-enzymatic glucose sensor, alkaline solution, differential pulse voltammetry

## Abstract

A cuprous oxide (Cu_2_O) thin layer served as the base for a non-enzymatic glucose sensor in an alkaline medium, 0.1 NaOH solution, with a linear range of 50–200 mg/dL using differential pulse voltammetry (DPV) measurement. An X-ray photoelectron spectroscopy (XPS) study confirmed the formation of the cuprous oxide layer on the thin gold film sensor prototype. Quantitative detection of glucose in both phosphate-buffered saline (PBS) and undiluted human serum was carried out. Neither ascorbic acid nor uric acid, even at a relatively high concentration level (100 mg/dL in serum), interfered with the glucose detection, demonstrating the excellent selectivity of this non-enzymatic cuprous oxide thin layer-based glucose sensor. Chronoamperometry and single potential amperometric voltammetry were used to verify the measurements obtained by DPV, and the positive results validated that the detection of glucose in a 0.1 M NaOH alkaline medium by DPV measurement was effective. Nickel, platinum, and copper are commonly used metals for non-enzymatic glucose detection. The performance of these metal-based sensors for glucose detection using DPV were also evaluated. The cuprous oxide (Cu_2_O) thin layer-based sensor showed the best sensitivity for glucose detection among the sensors evaluated.

## 1. Introduction

An abnormal glucose concentration level is directly related to diabetes, obesity, hyperglycemia, and encephalopathy [1]. Therefore, a cost-effective, accurate, and consistent glucose sensor is important in medical diagnosis. Measuring glucose concentration is also important for bio-processing and bio-reactor applications, as well as for home care usage. Current glucose sensors are typically based on an enzymatic mechanism with the advantages of low cost and simple operation; glucose oxidase immobilized on nanoparticles has also shown promising ability for the detection of glucose [2,3,4,5,6]. However, an enzyme-based biosensor is limited in accuracy and the reproducibility of measurements is only fair due to the loss of enzyme activity over time. Furthermore, the enzyme also has a limited active life time, which affects the manufacturing and shelf-life of the glucose sensor. Hence, a cost-effective, high-accuracy, and highly reproducible non-enzymatic glucose sensor is desirable for medical applications and bio-processing involving glucose as a reactant or product.

The oxidation of glucose can be detected electrochemically. Metal oxides, polymer films, and noble metals have been used as a substrate or catalyst for glucose oxidation reaction in non-enzymatic glucose sensor development [4,5,6,7]. Low sensitivity, complex sensor preparation procedures, and unpracticality for real usage were the major drawbacks. In this study, copper oxide was used for the detection of glucose in both phosphate-buffered saline (PBS) and human serum. Metal oxides exhibit good electrochemical properties based on their relatively high reactivity and large specific area at the nanoscale. Cuprous oxide is a metal oxide, which is a p-type semiconductor with a band gap of 1.9 eV. The high hole mobility and excess of oxygen atoms in the cuprous oxide structure demonstrate its high conductivity for electrochemical technique applications [8]. However, most of the synthesis and deposition processes for copper oxides are elaborate and tedious, requiring a high temperature and a relatively long operational time. In this study, the electrochemical deposition of cuprous oxide (Cu_2_O) was accomplished within a relatively short deposition time and with excellent reproducibility. The formation of Cu_2_O was accomplished using CuSO_4_·5H_2_O, lactic acid (C_3_H_6_O_3_), and NaOH, similar to the preparation approach described by Rahmen [9]. The deposited thin layer Cu_2_O was examined by X-ray photoelectron spectroscopy (XPS), and showed high reactivity and reproducibility for both the deposition process and electrochemical glucose detection.

In the presence of glucose and copper ions from the cuprous or cupric oxide, glucose was oxidized and the copper ions were reduced. The kinetics of the reaction mechanisms were not totally agreed upon, and the assessment of the actual kinetics of copper ions with glucose was beyond the scope of this presentation. Nevertheless, based on reported information of copper (II) oxide for oxidation reaction by hydroxide ions [10,11], copper (I) oxide did demonstrate a high conductivity.

In this study, Cu_2_O was deposited by electrochemical deposition on a thin gold film sensor prototype used in previous studies [12,13,14,15,16]. Glucose concentration ranges of 50–200 mg/dL in 0.1 M NaOH solution were detected using differential pulse voltammetry (DPV). Glucose detection was also performed in undiluted human serum using a minute quantity of 0.1 M NaOH and 3 µL serum containing glucose by DPV. Interference studies by uric acid and ascorbic acid showed that this Cu_2_O-based glucose sensor had good selectivity.

## 2. Materials and Methods

### 2.1. Apparatus and Reagents

Copper sulfate pentahydrate (#209198), lactic acid (#252476), sodium hydroxide (#306576), d-(+)-glucose (#G-8270), uric acid (#U-0881), l-ascorbic acid (#A5960), and human serum (#H3667) were obtained from Sigma Aldrich (St. Louis, MO, USA). Sodium chloride (#S-271) was purchased from Thermo-Fisher (Pittsburgh, PA, USA). Nickel chloride (#A0282977), and boric acid (#A0281874) were obtained from Acros Organics, Thermo-Fisher (Pittsburgh, PA, USA). A CHI 660C Electrochemical Workstation (CH Instrument, Inc., Austin, TX, USA) was used for the Chronoamperometry (CV) and DPV investigations. Other models of CHI 660 (Models A–E) could also be used. All experiments were conducted at room temperature. Scanning Electron Microscopy (SEM) was performed using a FEI HELIOS Nanolab 650 with a monochromated beam accelerating the electrons to 2 kV at a current of 13 nA. A secondary electron signal was used to capture the surface morphology. Time-of-flight secondary mass spectroscopy (Tof-SIMS) was used to capture the elemental distribution at the surface and as a function of depth by a PHI (Physical Electronics Inc., Chanhassen, MN, USA) Trift V nanoTOF. Thirty kilovolt (30 kV) pulsed Gallium primary Ions were used for spectroscopy and imaging and 3 kV Argon Ions for sputtering. X-ray photoelectron spectroscopy (XPS) was performed by a PHI Versaprobe 5000 Scanning X-Ray Photoelectron Spectrometer using an Aluminum Kα X-ray radiation (50 W, 15 kV, 1486.6 eV, 100 µm spot size on the sample) as the excitation source. The analyzer was operated at a constant pass energy of 23.5 eV. The calibration of the binding energy of the spectra was performed with the C 1s peak of the adventitious carbons, which was at 284.8 eV. The spectra of each sample were obtained for the C 1s, Cu 2p, and Cu LMM XPS regions. Spectrums were acquired at take-off angles of 0°, 45°, and 80° in order to obtain information about the composition as a function of the depth. The take-off angle is defined as the angle between the analyzer axis and the sample normal. Increasing it is equivalent to studying the photoelectrons that originate from a decreasing depth.

The electrochemical methods used in this study are described separately in Section 3 below.

### 2.2. Fabrication of Non-Enzymatic Cu_2_O Glucose Sensor

**Figure 1:**
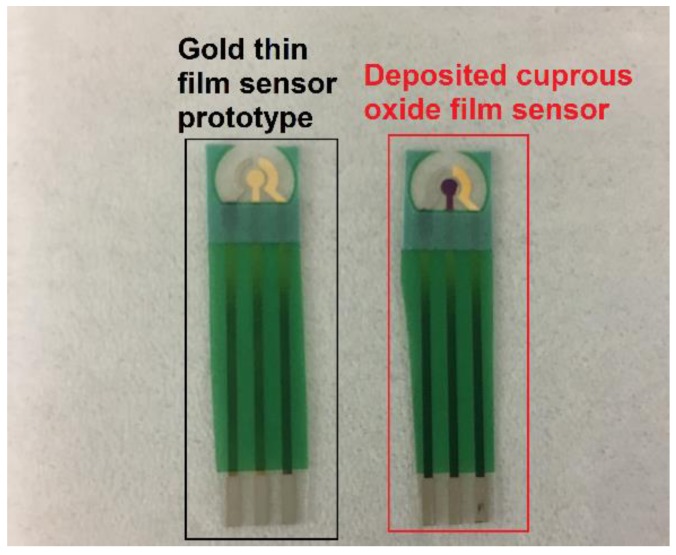
Comparison of gold sensor prototype and prepared cuprous oxide film sensor.

The sputtered thin gold film sensor prototype used in this study has been described elsewhere [12,13,14,15,16]. Electrochemical deposition of the cuprous oxide film on the thin gold film working sensor element was carried out. A mixture of 0.2 M of cupric sulfate, 3 M of lactic acid, and approximately 3 M of sodium hydroxide for adjusting the pH value to 12 was used as the electrolyte in this Cu_2_O deposition [9,17]. A water bath was used for maintaining the electrolyte solution at 40 °C. Twenty microliters (20 µL) of prepared electrolyte solution was cast onto the sensor, and linear sweep voltammetry was applied for the deposition of cuprous oxide. Linear sweep voltammetry of the potential from −0.8 V to −0.1 V was applied for deposition. The electrochemical deposition potential was at −0.36 V versus the thick-film printed Ag/AgCl reference electrode. The darker color of the working electrode (in the center of the sensor prototype) shown in Figure 1 shows the deposition of the cuprous oxide layer on the thin gold film working electrode. After deposition, the cuprous oxide thin film sensor was washed with deionized (DI) water, dried by nitrogen, and then ready for use.

## 3. Results and Discussion

### 3.1. Surface Characterization with SEM and Tof-SIMS

**Figure 2:**
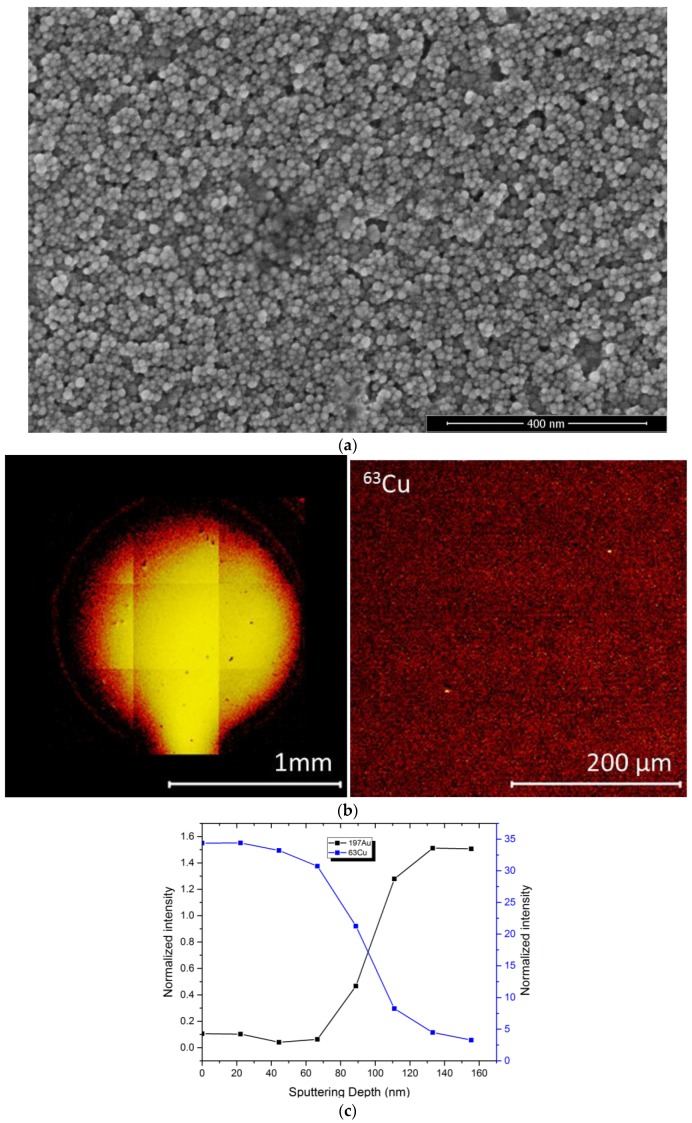
(**a**) SEM nanoparticle structure of cuprous oxide film; (**b**) TOF-SIMS evaluation of the homogeneity of the sensor’s surface; (**c**) Depth profile for the thickness of the cuprous oxide film.

The morphology and the homogeneity of the coating was examined with high resolution SEM imaging and TofSIMS chemical mapping (Figure 2a). The SEM images show a nanoparticle structure which is porous. Tof-SIMS chemical mapping (Figure 2b) also confirms the homogeneity of the coverage of the ^63^Cu^+^ signal from the entire sensor (image on the left) and magnified image in the center of the sensor (image on the right).

In order to evaluate the thickness of the coating, dual-beam depth profiling was performed on the Tof-SIMS in positive polarity to analyze the ^63^Cu^+^ and ^197^Au^+^ signals, which represented the coating and the substrate, respectively. The sputtering rate was calibrated on a 100 nm Ta_2_O_5_ on Ta substrate. This depth profile exhibited a thickness of approximately 100 nm as shown in Figure 2c.

### 3.2. Surface Characterization of Cuprous Oxide Layer with XPS

**Figure 3:**
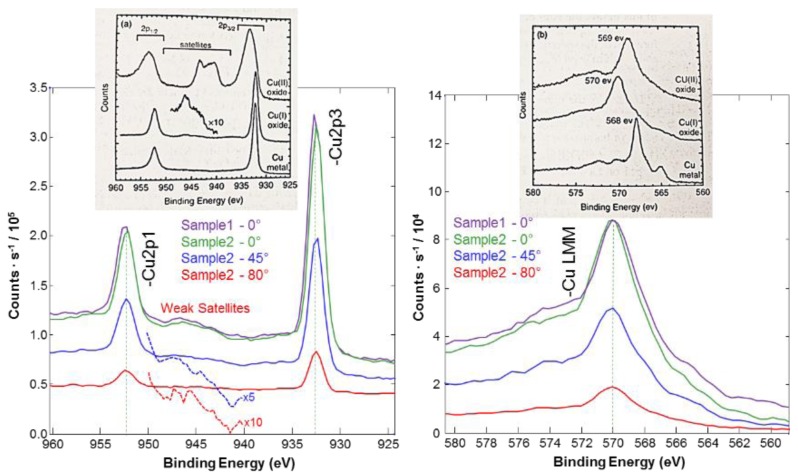
High resolution XPS spectrums of Copper photoelectron peaks compared on two different samples. The take-off angle on one of the samples verified the consistency of the measurement at different thicknesses.

X-ray photoelectron spectroscopy (XPS) was used for examining the formation of cuprous oxide film on the thin gold film working electrode. The chemical shift of the Cu 2p3/2 photoelectron peak was not detectable with the energy resolution of the XPS. The presence and the intensity of the satellite peaks along with the peak shape and position of Cu LMM were experimentally verified and matched with the reference data [18]. The experimental data from two different samples along with the reference data are shown in Figure 3. The decreasing of the take-off angle increased the height of the Cu 2p due to the sample facing the detector and with more photoelectrons reaching the detector. Consequently, the results also confirmed the presence of Cu_2_O by the formation of weak satellites in between the Cu 2p3/2 and Cu 2p1/2 peaks. This conclusion was supported by the spectra acquired at various take-off angles, proving that a uniform Cu_2_O layer was formed.

### 3.3. Electrochemical Measurement of Glucose by Differential Pulse Voltammetry (DPV)

**Figure 4:**
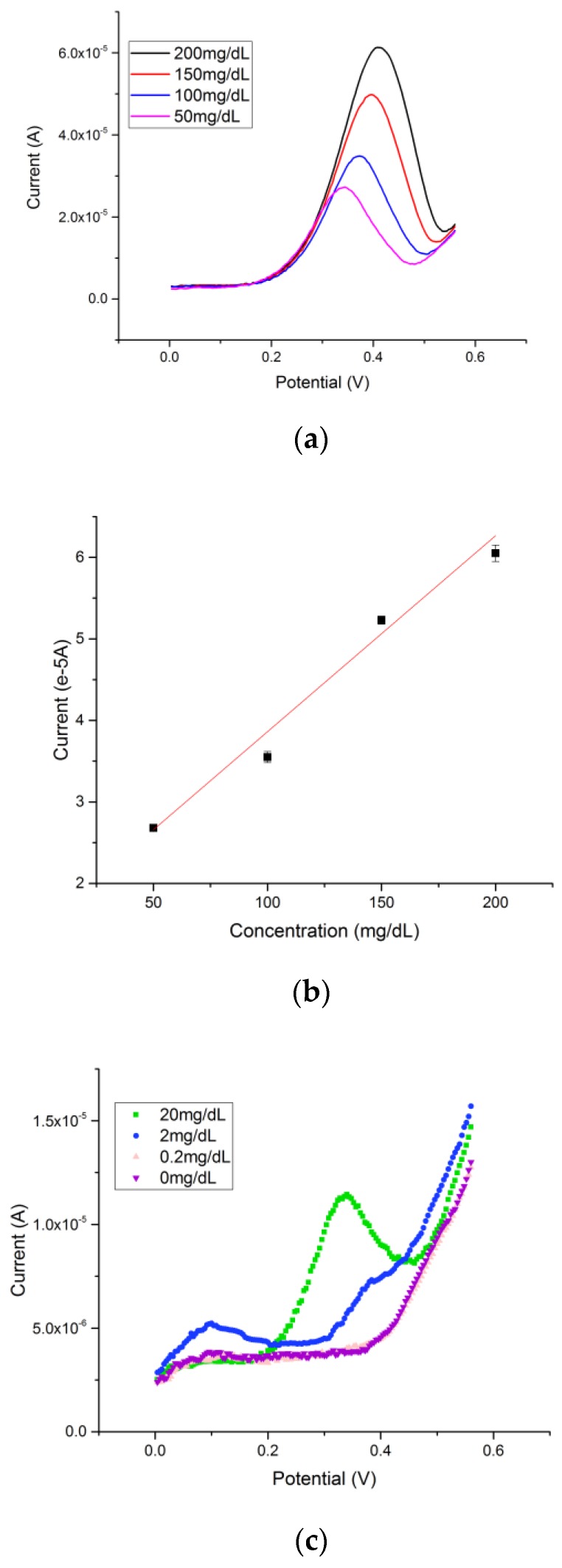
(**a**) Differential pulse voltammetry (DPV) measurements of glucose concentrations ranging from 200 mg/dL to 50 mg/dL; (**b**) Calibration linear relationship of DPV current outputs and concentrations of glucose; (**c**) Detection limit of the cuprous oxide sensor by using DPV was 0.2 mg/dL.

Glucose was prepared in a 0.1 M NaOH solution with the concentration ranging from 50 mg/dL to 200 mg/dL. Based on the mechanism of reaction of glucose with cuprous oxide in alkaline solution, an increase of the anodic peak current at a potential of approximately +0.4 V versus a thick-film printed Ag/AgCl reference electrode was observed with increasing concentration of glucose. In a typical experimental run, 20 µL of 0.1 M NaOH with a known glucose solution was drop-casted onto the cuprous oxide-based sensor. A rest time was set for 10 s, which allowed the hydroxide ion to first oxidize the cuprous oxide. DPV was then conducted in the range of 0 to +0.75 V versus the thick-film printed Ag/AgCl reference electrode. The DPV measurements of different glucose concentrations in 0.1 M NaOH solution are shown in Figure 4a. The calibration curve based on the DPV current output and concentration of glucose is shown in Figure 4b. A linear relationship Y = 0.024X + 1.46 with an adjusted R square value of 0.978 (*n* > 5) was established, demonstrating excellent sensitivity and reproducibility of the cuprous oxide film-based glucose sensor. Lower glucose levels were tested in order to determine the detection limitation of glucose of the cuprous oxide sensor. The testing range was from 0 mg/dL to 20 mg/dL of glucose in alkaline solution; the signal produced by 0 mg/dL was superimposed with the signal produced by 0.2 mg/dL as shown in Figure 4c, indicating a detection limit of 0.2 mg/dL of glucose by using differential pulse voltammetry.

### 3.4. Glucose Detection by Chronoamperometry (CA) and Single-Potential Amperometric Voltammetry 

**Figure 5:**
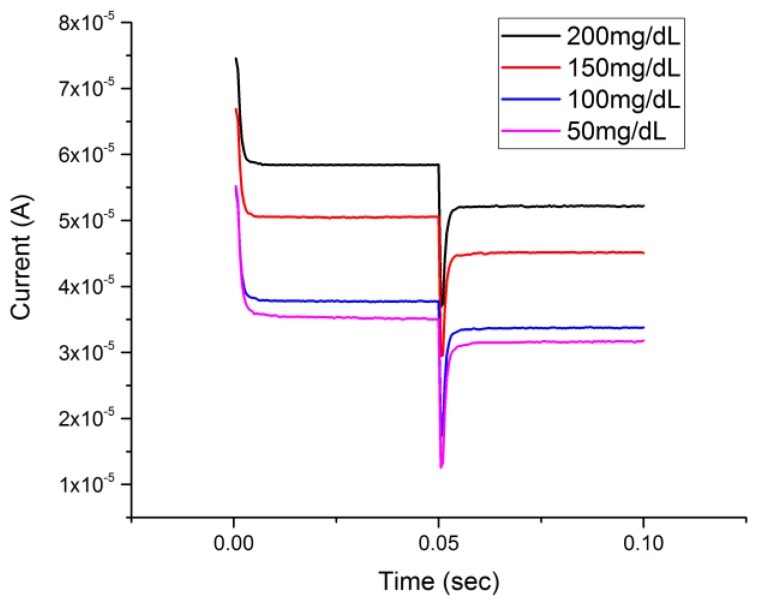
Chronoamperometry measurement of glucose concentrations ranging from 200 mg/dL to 50 mg/dL.

The determination of glucose using a Cu_2_O thin layer-based sensor and DPV measurement demonstrated that the detection technique was very effective. However, considering the electrochemical complexity of DPV compared to the commonly used CA and single-potential amperometric voltammetry, glucose detection by CA and single-potential amperometric voltammetry was also carried out. The results validated that the Cu_2_O thin layer-based sensor for glucose detection with different electrochemical detection techniques in addition to DPV measurement was successful. Furthermore, the results of this study verified the effectiveness of a Cu_2_O thin layer-based sensor for glucose detection in an alkaline medium. Figure 5 shows the chronoamperometry (CA) response of a Cu_2_O thin layer-based sensor to different glucose concentrations in a 0.1 M NaOH test medium. The testing procedure was the same as that mentioned in Section 3.2. In this CA measurement, a voltage of +0.35 V versus the Ag/AgCl reference was applied and then with a step change of potential to +0.4 V in voltage.

**Figure 6:**
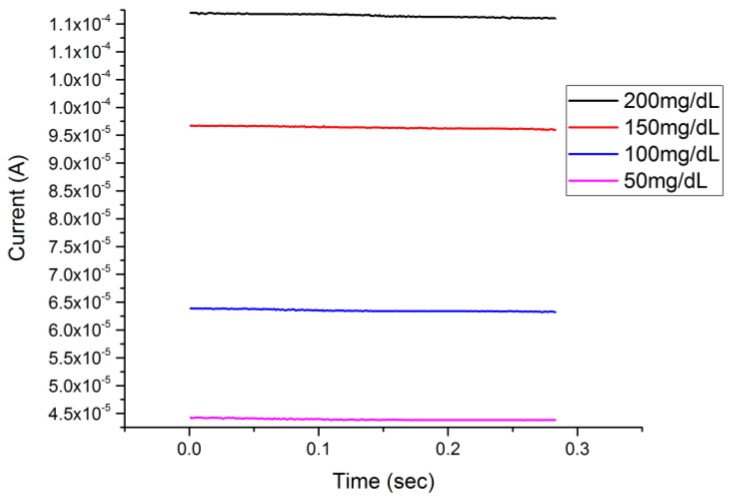
Single-potential amperometric voltammetry measurement of glucose concentrations ranging between 50 mg/dL and 200 mg/dL.

Figure 6 shows the single-potential amperometric voltammetry responses on glucose concentrations ranging from 50 mg/dL to 200 mg/dL in 0.1 M NaOH solution. There was no potential step change in single-potential amperometric voltammetry compared to the CA measurement. Thus, this cuprous oxide film-based sensor also showed an excellent response in the single-potential amperometric voltammetry at a single potential of +0.5 V versus the Ag/AgCl reference electrode. A rest time of 10 s was used to allow the reaction between the glucose and Cu_2_O to reach a steady state.

The detection responses of both chronoamperometry and single-potential amperometric voltammetry as demonstrated in Figure 5 and Figure 6 provided verification of the detection of glucose of a Cu_2_O thin layer-based sensor in an alkaline medium by the DPV technique. The performance of the cuprous oxide sensor was compared with our previous study, in which a micro-plotter printed cupric oxide sensor was developed. The response times of the detection in this study by DPV, CA, and single-potential voltammetry were 25 s, 0.05 s, and 0.3 s, respectively, which were shorter compared to the response times obtained in our previous studies. The average sensitivity calculated from three different voltammetries was 660 µA∙cm^−2^∙mM^−1^, which was lower than that of the previous study using a cupric oxide sensor for glucose measurement with cyclic voltammetry [14], but the higher reproducibility based on a higher R-square value for the related range of detection proved the reliability of the cuprous oxide sensor presented in this study.

### 3.5. Detection of Glucose in Undiluted Human Serum by DPV

**Figure 7:**
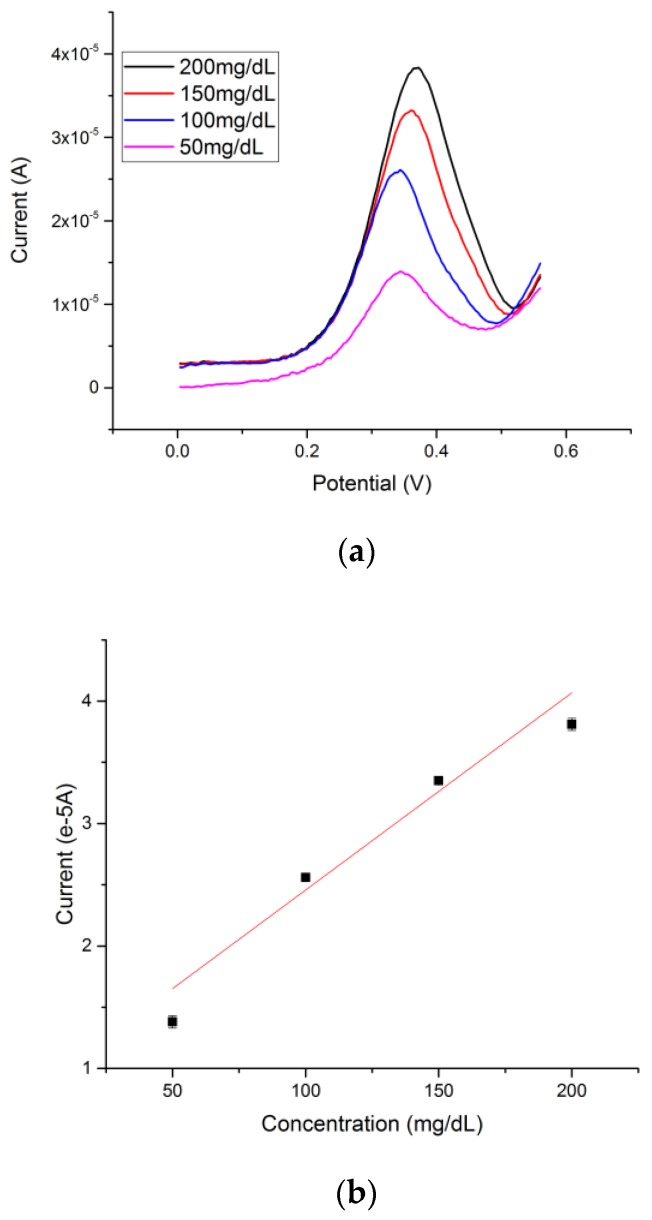
(**a**) Differential pulse voltammetry measurement of glucose concentrations ranging from 50 mg/dL to 200 mg/dL in undulated human serum; (**b**) Calibration linear relationship of DPV current outputs and concentrations of glucose.

In a typical run, 3 µL of glucose in serum solution was mixed with 3 µL of 0.1 M NaOH solution. Then, this 6 µL of the mixed solution was placed on the sensor and DPV was applied as described in Section 3.2. Figure 7a shows the DPV detection responses of the sensor for glucose solutions in human serum ranging from 50 mg/dL to 200 mg/dL. Figure 6b shows the calibrated linear fit for the DPV results with an equation of Y = 0.016X + 0.847 and an adjusted R-square value of 0.929 (*n* > 5).

The DPV measurements demonstrated that this Cu_2_O thin layer-based glucose sensor could be used effectively in blood serum by adding a minor volume of 3 µL of 0.1 M NaOH to the glucose test sample. This 3 µL of 0.1 M NaOH, or a similar hydroxide ion-containing solution, can be applied to the glucose test sample in serum with minimum inconvenience.

### 3.6. Interference Study of the Cu_2_O Thin Layer-Based Sensor for Glucose Detection 

**Figure 8:**
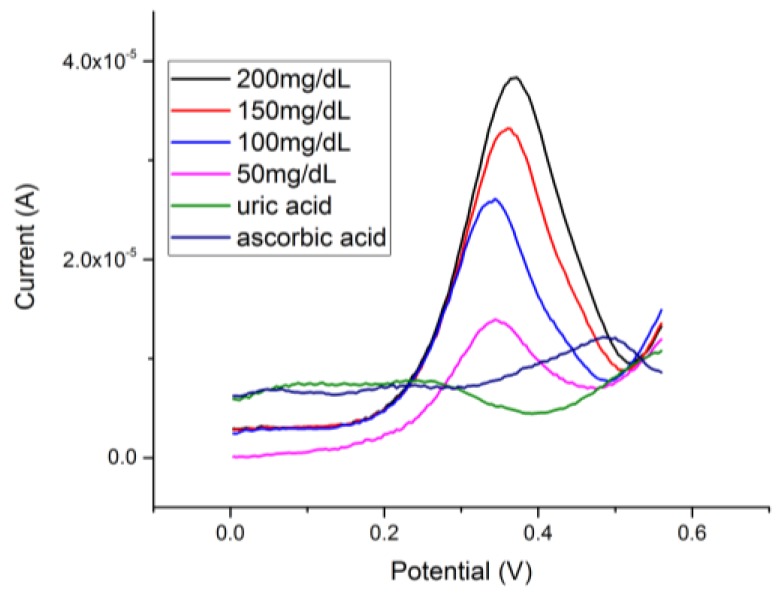
Interference tests from uric acid and ascorbic acid performed by DPV.

Interference testing was important to ensure the selectivity of the Cu_2_O thin layer-based sensor for glucose detection. Two common interference chemicals of glucose sensing, ascorbic acid and uric acid, were used in this study. The actual quantities of these interfering species are relatively minute compared with the quantity of glucose in human blood [19]. However, a relatively large quantity of ascorbic acid or uric acid was used in this interference study, demonstrating that the selectivity of this Cu_2_O thin layer-based glucose sensor was excellent. Ascorbic acid (100 mg/dL) and uric acid (100 mg/dL) were prepared individually in undiluted human serum. The same testing protocol described in Section 3.4 was conducted. Both ascorbic acid and uric acid at this high concentration level did not contribute any current in the DPV measurement of glucose detection as shown Figure 8. The results demonstrated that the selectivity of this Cu_2_O thin layer-based sensor for glucose sensing in an alkaline medium was excellent.

### 3.7. Non-Enzymatic Metallic Catalyst-Based Glucose Sensors in Alkaline Solution

In additional to cuprous oxide thin film, metallic catalysts, such as nickel, platinum, and copper, have also shown promising ability in reaction with glucose in an alkaline condition [19,20,21]. The reaction mechanism demonstrated that the alkaline solution oxidized the metal catalyst, then glucose reduced the oxidized metal producing gluconic acid [14,19,21]. Thus, the performance of metallic catalysts, including nickel, platinum, and copper, for the detection of glucose in alkaline solution was examined and compared with the cuprous oxide thin film sensor.

#### 3.7.1. Electrochemically Deposited Copper Film for the Detection of Glucose in Alkaline Solution

**Figure 9:**
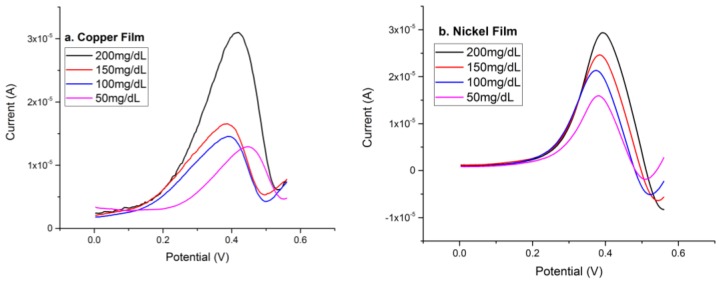

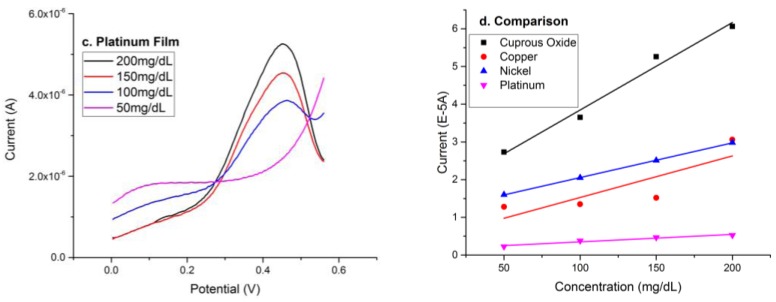
Detection response based on different metal films (**a**) copper, (**b**) nickel, (**c**) platinum, (**d**) comparison.

Copper has been used for the detection of organic compounds based on its oxidation activity in alkaline solution [21], including using cuprous and cupric oxides as discussed above. In order to assess the effect and the role of copper serving as a metal-based sensor for glucose detection, copper was electrochemically deposited onto the thin gold film sensor prototype and evaluated. Cathodic reduction of Cu^+2^ ions from an electrolyte was employed for the electrochemical deposition of copper onto the thin gold film sensor prototype. Typically, an electrolyte of 0.05 M of CuSO_4_ and 0.1 M of H_2_SO_4_ in aqueous solution was used for the copper deposition. Twenty microliters (20 µL) of the electrolyte was placed on the gold thin film sensor and linear sweep voltammetry of −0.9 V to −0.3 V was applied for the deposition of copper at room temperature. A cathodic peak current at −0.86 V versus the Ag/AgCl reference electrode was observed for this reduction reaction. The protocol for detecting glucose using this copper-based sensor was identical to the evaluation of other metal- or metal catalyst-based glucose sensors as described in Section 3.2. DPV was applied, and the oxidation reaction between the glucose and copper took place at around +0.40 V versus the Ag/AgCl reference electrode. The detection response time was 25 s. Figure 9a shows the anodic currents of the DPV measurements of glucose concentrations of 50–200 mg/dL in a 0.1M NaOH solution. The anodic peak currents appeared at approximately +0.40 V versus the thick-film printed Ag/AgCl reference electrode.

#### 3.7.2. Electrochemically Deposited Nickel Film for the Detection of Glucose in Basic Solution

Nickel is a good biological reaction catalyst with significant chemical activity [20]. Nickel is an active material for glucose detection in the presence of OH^−^ [21,22]. The performance of nickel on the detection of glucose was evaluated and compared to the cuprous oxide thin film layer-based sensor for glucose detection. Nickel was deposited electrochemically onto the thin gold film sensor prototype. An electrolyte containing 0.14 M NiCl_2_, 1 M NaCl, 0.5 M H_3_BO_3_, and a copious amount of HCl for adjusting the solution pH value to around 1.5 was prepared for the deposition of nickel [23]. Twenty microliters (20 µL) of an electrolyte was placed on the gold thin film sensor and linear sweep voltammetry of −1.2 V to −0.7 V was applied for the deposition of nickel at room temperature. An increasing reduction cathodic deposition peak was observed at −1 V versus the Ag/AgCl reference electrode. The procedure for the detection of glucose in an alkaline solution using this nickel-based sensor was identical to the process described in Section 3.2. Differential pulse voltammetry was applied, and an anodic peak was obtained at +0.38V versus the Ag/AgCl reference electrode [13,14,16], which was also confirmed by Luo’s study [21]. The detection response was 25 s. Figure 9b shows the differential pulse voltammetry graph for the detection of glucose in 0.1 M NaOH solution using the electrochemically deposited nickel thin film sensor covering the glucose concentration range of 50 mg/dL to 200 mg/dL.

#### 3.7.3. Sputtered Thin Platinum Film Sensor for the Detection of Glucose in Alkaline Solution

Platinum is well-accepted as a bioactive metal or catalyst for the detection of organic compounds, including carbohydrates, amino acids, and glucose [21]. Therefore, the detection of glucose using a platinum-based sensor was also studied in this research endeavor. The fabrication of the thin platinum film sensor prototype was identical to the process for the fabrication of the thin gold film sensor prototype [12,13,14,15,16], with the only difference that platinum (50 nm thickness) was used for the working and the counter electrodes instead of gold. Sputtering physical vapor deposition, laser ablation, and thick-film printing technologies were used, and the platinum thin film sensor could also be fabricated by a roll-to-roll cost-effective manufacturing process. The testing protocol of glucose using this platinum thin film-based sensor was identical to the testing procedure described in Section 3.2. DPV was applied, and anodic peak currents for different glucose concentrations were observed at approximately +0.43 V versus the Ag/AgCl reference electrode. The detection response was 25 s. Figure 9c shows the DPV measurements of various glucose concentrations of glucose in 0.1 M NaOH solution ranging from 50 mg/dL to 200 mg/dL.

This Section 3.7 discussed the experimental performance of various metals as catalysts for glucose detection using DPV measurements. Figure 9d summarized the performance of these non-enzymatic glucose sensors by displaying their calibration curves based on the data acquired. The sensitivity of each film was calculated as 119.3 µA cm^−2^ mmol^−1^ for copper, 99.6 µA cm^−2^ mmol^−1^ for nickel, and 21.4 µA cm^−2^ mmol^−1^ for platinum. Compared with the metal film sensors for the detection of glucose in alkaline solution, cuprous oxide demonstrated the highest current outputs and the better sensitivity, showing promise for application as a non-enzymatic glucose sensor.

## 4. Conclusions

A non-enzymatic cuprous oxide (Cu_2_O) thin layer-based sensor for the detection of glucose in an alkaline medium, 0.1 NaOH solution, over the glucose concentration range of 50–200 mg/dL was successfully developed using differential pulse voltammetry (DPV) measurement. X-ray photoelectron spectroscopy (XPS) confirmed the formation of a cuprous oxide, Cu_2_O, layer on the thin gold film sensor prototype. The evaluation of glucose in both phosphate-buffered saline (PBS) and undiluted human serum was carried out. The 0.1 M NaOH alkaline solution used was minute, 3 µL in a total of 6 µL test medium. Neither ascorbic acid nor uric acid, even at a high concentration level (100 mg/dL in serum), interfered with the cuprous oxide (Cu_2_O) thin layer-based sensor in the glucose measurement, demonstrating that the selectivity of this non-enzymatic cuprous oxide (Cu_2_O) thin layer-based sensor was excellent. Chronoamperometry (CA) and single-potential amperometric voltammetry were also used in the glucose detection experiments using this cuprous oxide (Cu_2_O) thin layer-based sensor. The positive results verified the validity of detecting glucose in a 0.1 M NaOH alkaline medium by DPV measurement. Nickel, platinum, and copper are commonly used metals for non-enzymatic glucose detection. The performance of these metal based sensors for glucose detection using the DPV technique was experimentally evaluated. The cuprous oxide (Cu_2_O) thin layer-based sensor showed the best sensitivity for glucose detection among the sensors evaluated.
